# Anti-platelet aggregation of Panax notoginseng triol saponins by regulating GP1BA for ischemic stroke therapy

**DOI:** 10.1186/s13020-021-00424-3

**Published:** 2021-01-19

**Authors:** Zhi-yi Xu, Yang Xu, Xiao-fang Xie, Yin Tian, Jun-hui Sui, Yong Sun, Da-sheng Lin, Xing Gao, Cheng Peng, Yu-jiang Fan

**Affiliations:** 1 National Engineering Research Center for Biomaterials, Sichuan University, 29 Wangjiang Road, Chengdu, 610064 Sichuan China; 2 School of Pharmacy, Chengdu University of Traditional Chinese Medicine, Liutai Avenue NO. 1166, Wenjiang District, Chengdu, 611137 Sichuan China; 3 Chengdu Huasun Technology Group Inc., Ltd, Shuxin Avenue No.1168, Western Hi-tech Zone, Chengdu, 611731 Sichuan China

**Keywords:** PTS, Anti-platelet aggregation, GP1BA, Binding affinity, Ischemic stroke

## Abstract

**Background:**

Panax notoginseng triol saponins (PTS) has been used clinically for ischemic stroke therapy (IST) in China for more than 17 years due to its anti-platelet aggregation and neuro-protective effects, but its mechanism of action is not fully understand. In this study, anti-platelet aggregation-related protein analysis and computer simulations of drug-protein binding interactions were performed to explore the mechanism of the effects of PTS against ischemic stroke in an ischemia reperfusion model.

**Methods:**

Three oral doses of PTS were administered in a model of middle cerebral artery occlusion (MCAO) in rats. Panax notoginseng total saponins (PNS) and a combination of PTS and aspirin were chosen for comparison. To evaluate therapeutic effects and explore possible mechanisms of anti-platelet aggregation, we measured cerebral infarct size and water content in brain tissue, histomorphological changes, expression of related factors (such as arachidonic acid metabolites) and platelet receptors in serum, as well as the binding affinity of PTS for platelet adhesion receptors.

**Results:**

Compared with PNS, PTS showed a stronger and more potent anti-platelet aggregation effect in MCAO model rats. The combination of PTS and aspirin could reduce adverse gastrointestinal effects by regulating the TXA_2_/PGI_2_ ratio. We demonstrated for the first time that PTS was able to regulate Glycoprotein Ib-α (GP1BA) in a model animal. The binding of ginsenoside Rg_1_ and GP1BA could form a stable structure. Moreover, PTS could reduce von Willebrand factor (VWF)-mediated platelet adhesion to damaged vascular endothelium, and thus enhance the probability of anti-platelet aggregation and anti-thrombosis under pathological conditions.

**Conclusions:**

Our results showed that GP1BA was closely related to the anti-platelet aggregation action of PTS, which provided new scientific and molecular evidence for its clinical application.
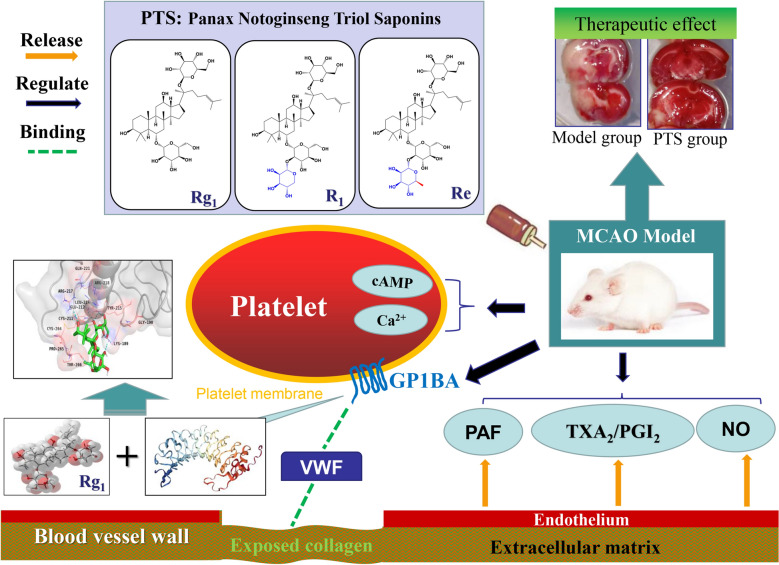

## Background

Epidemiological studies show that stroke is the leading cause of death after heart disease with clinical features such as high morbidity, high recurrence rate, and high disability rate [1]. China has the highest incidence of ischemic stroke and the number of new stroke patients is increasing by 1.5 to 2 million per year, which has become a major health threat to residents and a serious economic and psychological burden on patients and their families [2, 3]. Therefore, the prevention and improvement of ischemic stroke has become an urgent issue in clinical medicine.

The formation of intravascular thrombosis leading to vascular stenosis and the further formation of cerebral artery obstruction are the main causes of ischemic stroke. While platelet plays a key role in the pathogenesis of ischemic stroke, and their degree of activation is a key factor in determining the formation and development of thrombosis to a certain extent, they also affect the reperfusion process as well. Thrombosis exacerbates effects such as oxidative stress, cell apoptosis, and inflammatory cytokine release and causes ischemia–reperfusion injury [4, 5].

Modern pharmacological studies have shown that the arachidonic acid pathway, adenosine diphosphate pathway, and platelet activation factor pathway are three main pathways of platelet activation aggregation through different mechanisms. Among them, arachidonic acid is metabolized by cyclooxygenase into thromboxane A2 (TXA_2_), which induces platelet aggregation, whereas the adenosine diphosphate and platelet-activating factor pathways activate phospholipase C through specific receptors acting on the surface of the cell membrane to promote the release of Ca^2+^ from calcium reservoirs, which induces platelet and leukocyte adhesion, aggregation, and release [6]. Currently, the representative anti-platelet agents, including aspirin, clopidogrel, and dipyridamole have been widely used in the prevention and treatment of stroke, and the clinical efficacy of these agents has been widely confirmed. As a cyclooxygenase inhibitor, aspirin can irreversibly inhibit cyclooxygenase and simultaneously promote the inability of arachidonic acid to be converted into intracyclic peroxide in platelets. Thus, aspirin can inhibit the formation of TXA_2_, effectively dilating blood vessels, and further exert anti-platelet aggregation and antithrombotic effects [7]. However, according to clinical statistics, adverse responses to aspirin occur in 5% to 45% of patients, which is an independent risk factor for gastrointestinal damage and poor prognosis of coronary heart disease, as well as the main reason for poor medication compliance and even withdrawal [8]. Therefore, the use of aspirin in the treatment of ischemic stroke is extremely limited by the potential clinical risks.

Panax notoginseng, as the representative traditional herbal drug for promoting blood circulation, is widely used in treating ischemic stroke in China. Saponins are recognized as the active constituents and Q-marker of panax notoginseng, and are mainly divided into diol and triol saponins [9]. Increasingly, research suggests that Panax notoginseng total saponins (PNS) can affect the cardiovascular system via diverse mechanisms of action to prevent the formation of foam cells, inhibit inflammation, suppress cell proliferation/migration, protect cerebral/myocardial cells and regulate lipid metabolism as well as blood coagulation [10]. Moreover, clinical evidence from China shows that PNS appears to be effective in treating acute cerebral infarction [11]. Although several reports have revealed that a monomeric compound in PNS exhibited notable therapeutic activities, studies have not determined which type of saponins of PNS is most effective.

Panax notoginseng triol saponins (PTS) are the active pharmaceutical ingredients of Sanqi Tongshu capsule which has been on the market in China since 2003 for ischemic stroke therapy. Ginsenoside Rg_1_, ginsenoside Re and notoginsenoside R_1_ are the main components of PTS with a total content of more than 67%, the remainder consists of small amounts of other saponins, flavonoids, polysaccharides, and amino acids. PTS has obvious cerebral protective effects in animal models of cerebral ischemia and cerebral infarction and has strong activity in reducing platelet aggregation and platelet adhesion. Also, PTS has a good therapeutic effect by improving outcomes after ischemic stroke as a safe complementary medicine to aspirin [12, 13]. Among the ginsenosides, Rg_1_ exhibits the strongest anti-platelet activity and can achieve anti-thrombosis by inhibiting platelet aggregation, reducing blood viscosity, enhancing fibrinolytic system activity, and promoting the release of vascular endothelial NO; R_1_ can inhibit the production of peroxides and leukocyte adhesion; Re can enhance adenylate cyclase activity, Ca^2+^ levels of platelet cell, and inhibit platelet activation [14, 15]. The clinical efficacy of PTS has been demonstrated for many years; however, the mechanism of action of PTS is not sufficiently understood, especially with regard to the systematic and in-depth interpretation of targets and pathways related to anti-platelet aggregation, which affects the current clinical value of the drug.

Platelets can adhere to collagen on the vascular wall, and thus cause the release of inducers and activate aggregation to form thrombus, which involves a variety of endogenous substances such as proteins and signal factors in the body. Studies have shown that during the initial adhesion of platelets, an important type of platelet membrane glycoprotein, platelet receptor glycoprotein Ib-α (GP1BA), can effectively combine with VWF and fibrinogen to form the glycoprotein Ib/IX/V complex and participate in platelet aggregation, which is a key step of thrombosis [16, 17]. Platelet membrane glycoprotein receptors can increase the rate of platelet aggregation, induce thrombus formation, and eventually cause ischemic stroke disease [18]. Thus, it is reasonable to believe that GP1BA may play a crucial role in thrombosis.

Based on the above research and discussions, we selected three doses of PTS (PTS-H, PTS-M and PTS-L) for oral administration in middle cerebral artery occlusion (MCAO) model rats [19, 20]. PNS and the combination of PTS and aspirin were chosen for comparison to evaluate therapeutic effects. We measured the cerebral infarct size and water content in brain tissue, histomorphological characteristics, expression of related factors and platelet receptors in serum, as well as the binding affinity of PTS to further investigate the mechanism of PTS on platelet aggregation for ischemic stroke therapy. Specifically, we investigated the effect of PTS and aspirin on arachidonic acid metabolism and the influence of PTS on binding of the glycoprotein Ib/IX/V complex and VWF. Furthermore, we conducted studies to determine the molecular mechanism involving PTS and specific proteins using surface plasmon resonance (SPR) [21] and molecular docking simulations [22].

## Materials and methods

### Materials

PTS (Lot No.: 20190318) was provided by Chengdu Huasun Technology Group (Chengdu, China). Aspirin enteric-coated tablets (Lot No.: BJ45216) were purchased from Bayer Health Care (Milano, Italy). Xuesaitong soft capsules (Lot No.: 20190322) with a 75% content of PNS (saponins extracted from notoginseng, including ginsenoside Rg_1_, ginsenoside Rb_1_, ginsenoside Re, notoginsenoside R_1_, and ginsenoside Rd) were purchased from Kunming Shenghuo Pharmaceutical (Kunming, China). Reference Standards of ginsenoside Rg_1_, ginsenoside Re and notoginsenoside R_1_ were purchased from the National Institutes for Food and Drug Control (Beijing, China). Toluidine Blue O was purchased from Scientan (Beijing, China). TTC, and other chemical reagents were purchased from Chengdu Chron Chemicals (Chengdu, China). All chemicals were of analytical grade and used as received. The kits for determining cAMP, TXA_2_, TXB_2,_ PGI_2_, and 6-keto-PGF1α were purchased from Elabscience Biotechnology (Wuhan, China). The platelet protein extraction kit was obtained from Bestbio Biotechnology (Shanghai, Chinese). The kits for determining NO and NOS were purchased from Nanjing KeyGen Biotech (Nanjing, China). The peripheral blood platelet separation kit was obtained from Haoyang Biological Manufacture (Tianjin, China). PAF was purchased from Abcam (Cambridge, USA). GP1BA was purchased from Proteintech (Rosemont, USA). The CM5 Chip, Peptide Coupling Kit, GST Capture Kit and other buffers used in the SPR test were purchased from GE Healthcare Life Sciences.

### Division of groups and the middle cerebral artery occlusion model

All research animals were provided by the Experimental Animal Center of Chengdu University of Traditional Chinese Medicine and raised in SPF-class housing in a laboratory with controlled conditions (20–22℃, relative humidity of 50–60%, and 12 h light–dark cycles). The animals were fed with water and a commercial rat pellet diet. All animal experiments were approved by the Sichuan Provincial Committee for Experimental Animal Management. After acclimation, testing drugs were orally administrated orally to the adult male SD rats (weighing 300 ± 20 g) at 10 mL/kg by gavage, once a day for 6 consecutive days. Then the animals were divided into seven groups each group contains 8 of rats, with different forms of administration: sham surgery and model control (distilled water), positive control (PNS at 28 mg/kg), high-, medium- and low-dose PTS (100, 50 and 25 mg/kg, respectively) as well as the combined treatment (PTS at 50 mg/kg and aspirin at 21 mg/kg). Specifically, the MCAO model of rats was established at 30 min after gavage on day 6, the operation of modeling was improved according to Longa’s method [23] as described below: the rats underwent fasting for a period of 12 h prior to the experimental procedures with water available ad libitum, and were anesthetized intraperitoneally using 10% chloral hydrate at 35 mg/100 g body weight. After the righting reflex disappeared, the animals were fixed in a supine position for neck fur removal and disinfection, and then incised at the midline of the neck. After the left common carotid artery (CCA) was located, the external carotid artery (ECA) and internal carotid artery (ICA) were separated while injury to vagus was avoided. Then the proximal CCA, and ECA and all its branches were ligated to separate the trunk. A fishing line with a diameter of 0.20 mm and a polished tip end was advanced into the ICA under direct vision through an incision which was made 4 mm from the bifurcation proximally. The insertion went to about (18.5 ± 0.5) mm till the proximal anterior cerebral artery, thus completely blocking the blood supply from the middle cerebral artery. Reperfusion was performed after 2 h of ischemia. At reperfusion, the nylon fishing line was gently withdrawn by a length of 10 mm. The intraoperative and postoperative room temperature was maintained at about 25 ℃, and the animals were irradiated using incandescent light to maintain their anal temperature at 37 ± 1 °C until recovery of activity. A model was determined to be successful based on the following criteria: (1) positive Horner’s syndrome on the right side and (2) hemiplegia on the left side mainly manifesting as forelimb movement abnormality. In the sham surgery group, all procedures were performed except there was no insertion of a fishing line. The animals were dosed twice in the process of establishing the model: 2 h after ischemia before reperfusion and 6 h after reperfusion, respectively. Then, the blood was taken from the femoral artery of the leg and the brains were collected after reperfusion for 22 h.

### Determination of cerebral infarct size and water content in brain tissue

After reperfusion for 22 h, the rats were decapitated and their rhinencephalon, lower brain stem, and cerebellum were removed. After weighing, coronal sections with a thickness of approximately 2 mm were obtained from the brain tissues and each side of the ischemic brain tissue was cut into five pieces. Then, the pieces of brain were immediately placed in a 2% TTC solution and incubated for 30 min in the dark at 37 °C. After staining, the normal brain tissue was rosy, whereas the cerebral infarct area was white. The total area and the infarcted area of the ischemic brain tissue in each slice were calculated by Nodus DanioScope Version 1.0.109. The percentage of the brain in the infarct area to the total brain was taken as the infarct size (%). The stained brain tissue was weighed before and after being dried in an oven at 105 °C for 48 h and the water content in the brain tissue was calculated according to the following formula:

$$\begin{aligned} &{\text{Water content in brain tissue }}\left( \% \right) \\ &\quad= 100\% .\left[ {1 - \frac{{{\text{dry}}\;{\text{weight}}\;{\text{of}}\;{\text{brain}}}}{{{\text{wet}}\;{\text{weight}}\;{\text{of}}\;{\text{brain}}}}} \right] \times 100\% \end{aligned}$$

### Histomorphological observations

Sections were obtained from the same area of the brain tissue for rats in each group. The tissue samples were fixed with 4% paraformaldehyde, embedded with paraffin, and then cut into pieces (4 µm) using a Leica RM2235 microtome. Subsequently, H&E and Nissl’s staining were implemented to observe histopathological changes using the microscopic imaging system. The number of Nissl bodies were quantitatively analyzed via Image Pro Plus 6.0 software and the evaluation was performed by a blinded investigator.

### Determination of related factors and platelet receptor expression

Blood samples were collected and centrifuged at 3500 rpm for 10 min to obtain serum and plasma to investigate the following: cAMP, Ca^2+^, NO, NOS, TXA_2_, TXB_2,_ PGI_2_ and 6-keto-PGF1α. cAMP, Ca^2+^, NO and NOS were determined using corresponding test kits and TXA_2_, TXB_2,_ PGI_2_ and 6-keto-PGF1α were determined using ELISA. Also, platelets were collected from the serum of the rats in each group using platelet isolation kits. Total protein was collected with a kit for the rest. GP1BA and PAF protein expression was determined by Western Blot.

### Methods for the SPR test

After coupling captured molecule and pre-concentration of ligand, we determined that the results indicated that GP1BA could be optimally captured at pH 5.0. The relevant parameters were set as follows: R_max_ = analyte Mw/ligand Mw × RL × Sm; anti-GST antibody: 26 kDa; GP1BA: 56.7 kDa; R_max_: typically, 100 RU; Sm: stoichiometric ratio, typically 1 (actual captured > RL); GP1BA: pI = 5.63, pH of sodium acetate buffer: 3.5 < pH < pI. The GP1BA and different concentrations of samples (Rg_1_, Re, R_1_) were prepared and injected for the test. The contact time and flow rate were set according to the system instructions. The data collected were analyzed and fitted by PLEXERA SPR Date Analysis Module (DAM). Kinetic data such as fitting constant, dissociation constant, and equilibrium dissociation constant were calculated based on curve fitting to obtain the specificity of molecular binding and the binding process.

### Method of molecular docking

The geometric structures of ginsenoside Rg_1_, Re, and notoginsenoside R_1_ were optimized using the density functional method [24, 25] at the B3LYP level of theory [26, 27] with the 6-31G(d) basis set in Gaussian 16 package. The 3D crystal structure of the human platelet receptor GP1BA was obtained from the RCSB Protein Data Bank (PDB ID: 1P9A) [28]. All docking simulations were performed with the Lamarckian genetic algorithm in Autodock 4.2 software [29]. The docking images were generated by PyMOL.

### Statistical analysis

Parametrical data were expressed as the mean ± standard deviations (SD). Statistical comparisons in all of the results except SPR test and molecular docking among groups were performed by one-way analysis of variance (ANOVA) with IBM SPSS for Windows (version 17.0, USA). The level of statistical significance was set at P < 0.05 or P < 0.01 by student *t-*test. The homogeneity test of variance was compared in each group. The least significant difference (LSD) test was conducted when the variance was homogeneous, and Tamhane’s T2 test was conducted when the variance was heterogeneous.

## Results

### In vivo therapeutic effect of PTS in the MCAO model

**Fig. 1:**
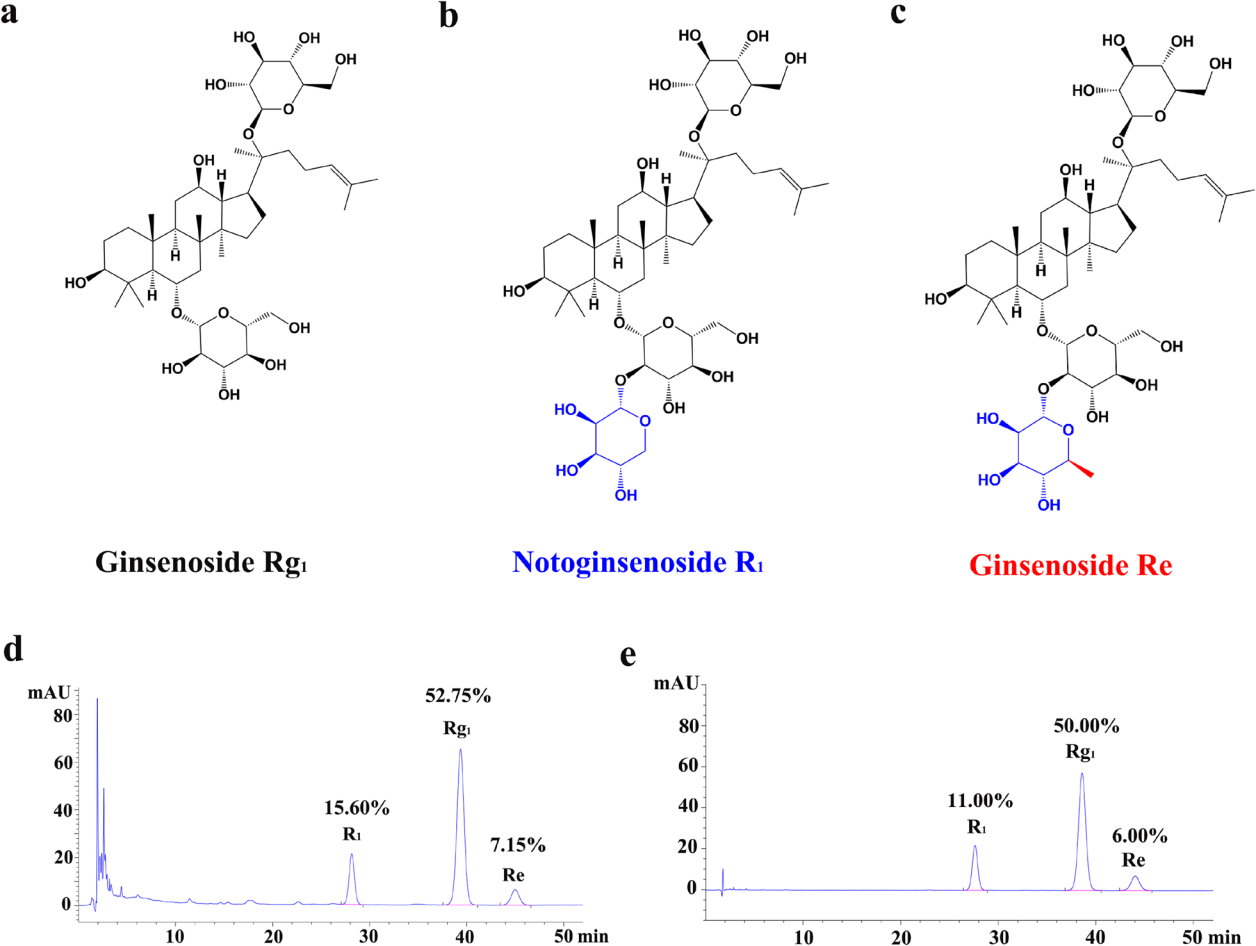
Chemical structures and representative HPLC chromatograms of PTS. 2D Chemical structure of **a** ginsenoside Rg_1_, **b** notoginsenoside R_1_ and **c** ginsenoside Re. **d** HPLC chromatogram of Panaxtriol Saponins (Lot No.:20190318) and the content of Rg_1_, R_1_ and Re. **e** HPLC chromatogram of reference standard and the percentage of each reference. Difference in structures of the above three compounds were shown in blue or red. The determination method was based on the Chinese pharmacopoeia 2015 and the absorbance at 210 nm was monitored by HPLC

**Fig. 2:**
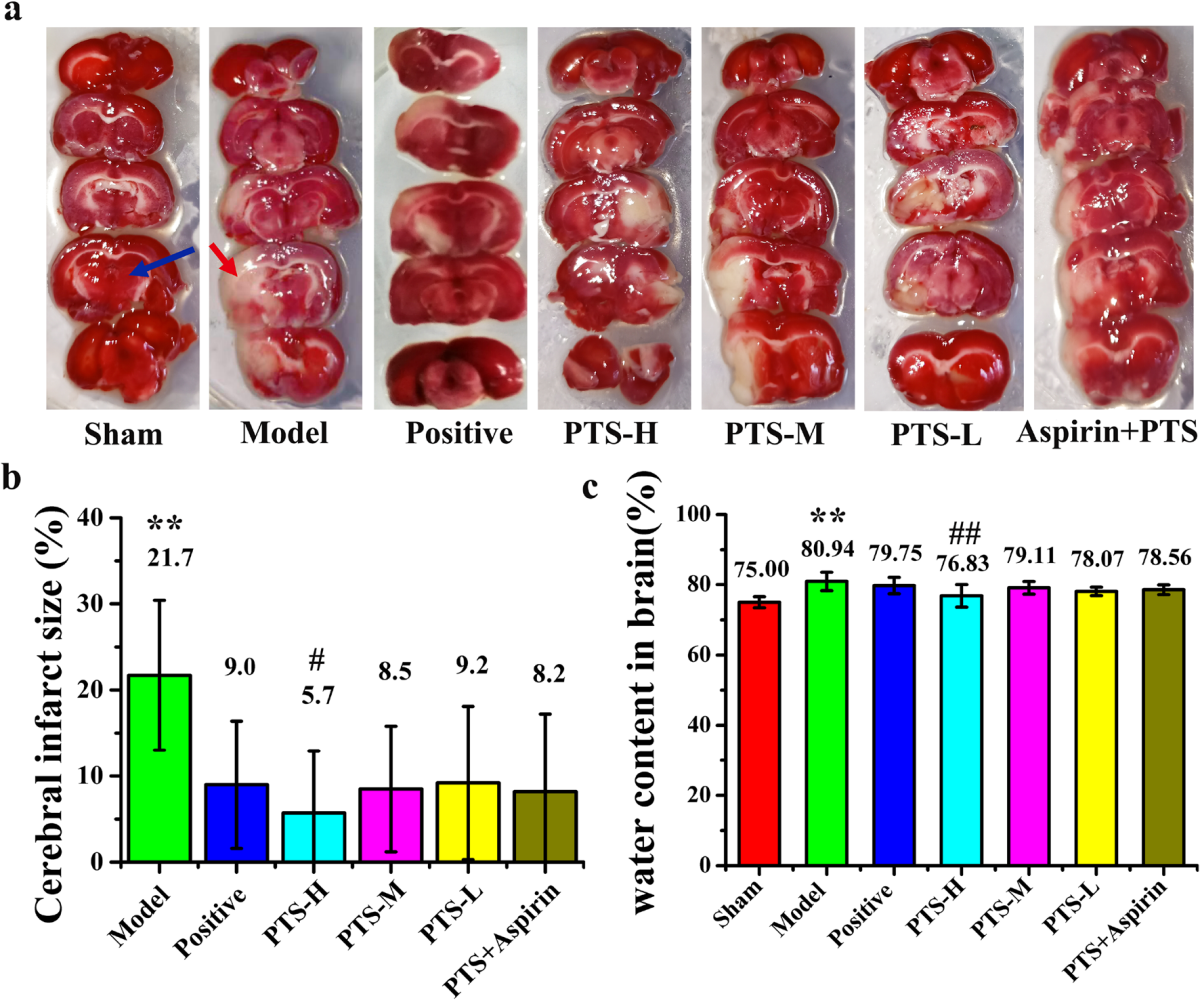
**a** TTC staining images of each group after reperfusion for 22 h. Normal brain tissue appears red as shown by the blue arrow, and infarct tissue appears pale gray as shown by the red arrow. Quantitative analysis of the **b** cerebral infarct size and **c** water content in brain of each group. Values are the mean ± SD (n = 8). Comparing model control group with sham surgery group, *P < 0.05, **P < 0.01; comparing treatment groups with model control group, ^#^P < 0.05, ^##^P < 0.01

The chemical structure of the main components of PTS is shown in Fig. 1a–c and the representative HPLC chromatograms of PTS and the reference standard sample is presented in Fig. 1d, e. The location of the chromatographic peak for the active ingredients in PTS (ginsenoside Rg_1_, ginsenoside Re and notoginsenoside R_1_) was consistent with the standard sample. Figure 2 shows the results of the animal experiments. Compared with the sham surgery group, the percentage of cerebral infarct size and water content in the brain tissue of the model control group was significantly changed (P < 0.01). The cerebral infarction size of each treatment group decreased in different degrees with the high-dose PTS group presenting the smallest size, which was significantly different from that of the model group (P < 0.05). Compared with the model group, the high-dose PTS group had a significant reduction in the brain water content (P < 0.01). These data indicate that high-dose PTS can efficiently reduce the size of the cerebral infarction and water content in brain tissue after ischemia–reperfusion. Compared with the middle-dose PTS, there was no significant difference between the combination of aspirin and PTS.

**Fig. 3:**
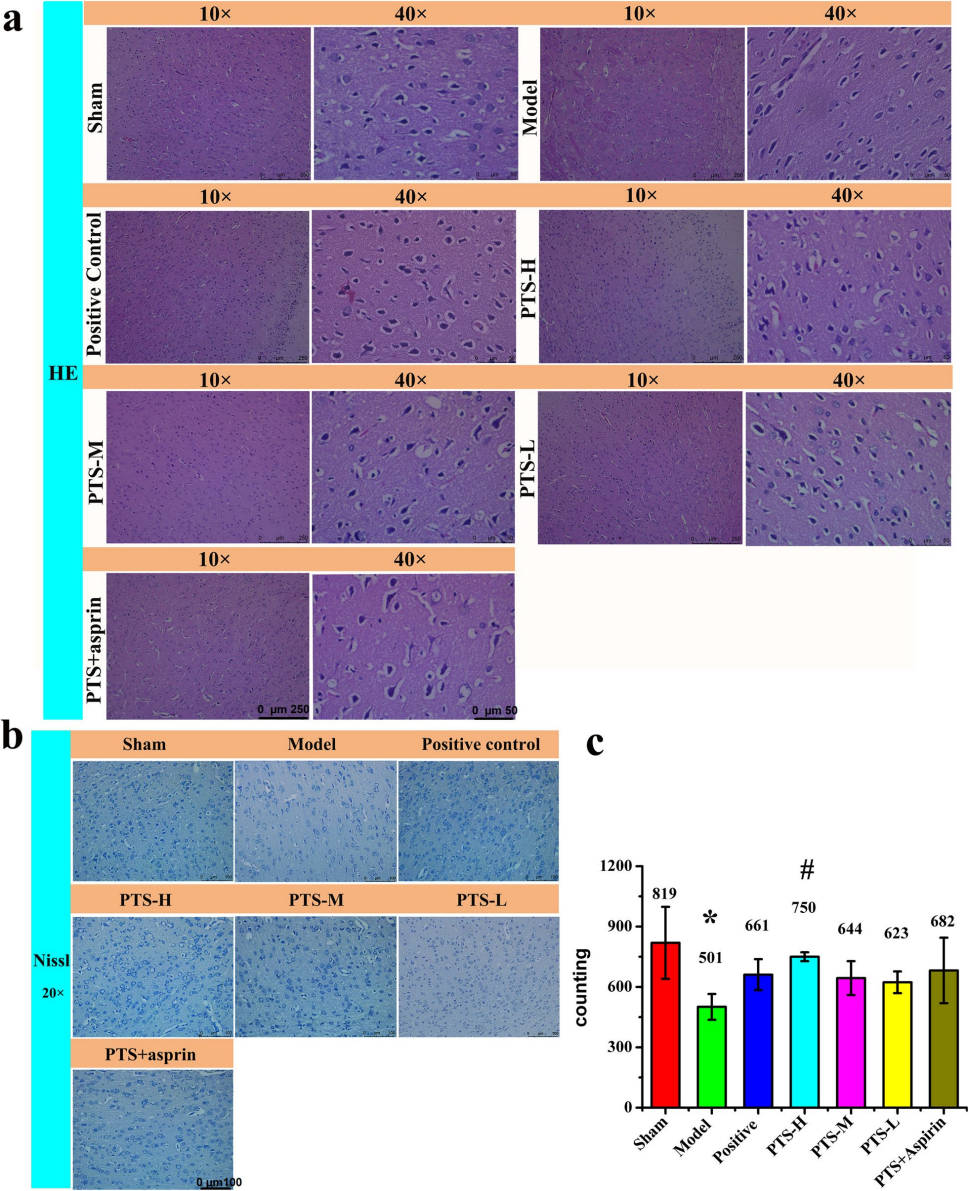
**a** H&E staining (in 10× and 40×) and **b** Nissl staining (in 20×) analysis of brain tissue in each group after reperfusion for 22 h. In H&E staining sections, neuronal pyramidal cell and its dendrite could be clearly observed, and central Nissl bodies were dissolved in red neurons. In Nissl staining, Nissl bodies were highlighted in dark blue. After modeling, neuronal cell degeneration and necrosis were observed and central Nissl bodies were dissolved, showing red neurons. **c** The number of Nissl bodies was analyzed by Image Pro Plus 6.0 software. Values are the mean ± SD (n = 6). Comparing model control group with sham surgery group, *P < 0.05; comparing treatment groups with model control group, ^#^P < 0.05

H&E staining was performed and the microscopy results are shown in Fig. 3a. In the model group, we observed necrosis and liquefaction of nerve tissue, and we also observed sieve reticular lesion, which clearly demarcated from the surrounding tissue, and a large number of nucleus fragments in the infarcted area and even condensation and fragmentation of the nucleus. Following drug treatment, we found that the brain histopathology of each group was improved to some extent. The Nissl staining was observed using a microscope and is presented in Fig. 3b, c, in which the Nissl bodies are highlighted with dark blue. The results revealed that a large number of Nissl bodies were destroyed in the model group, whereas the number of Nissl bodies increased obviously following drug treatment, especially in the high-dose PTS groups, where the number of Nissl bodies was similar to that of the sham group.

### PTS regulates platelet-associated factors in the MCAO model

**Fig. 4:**
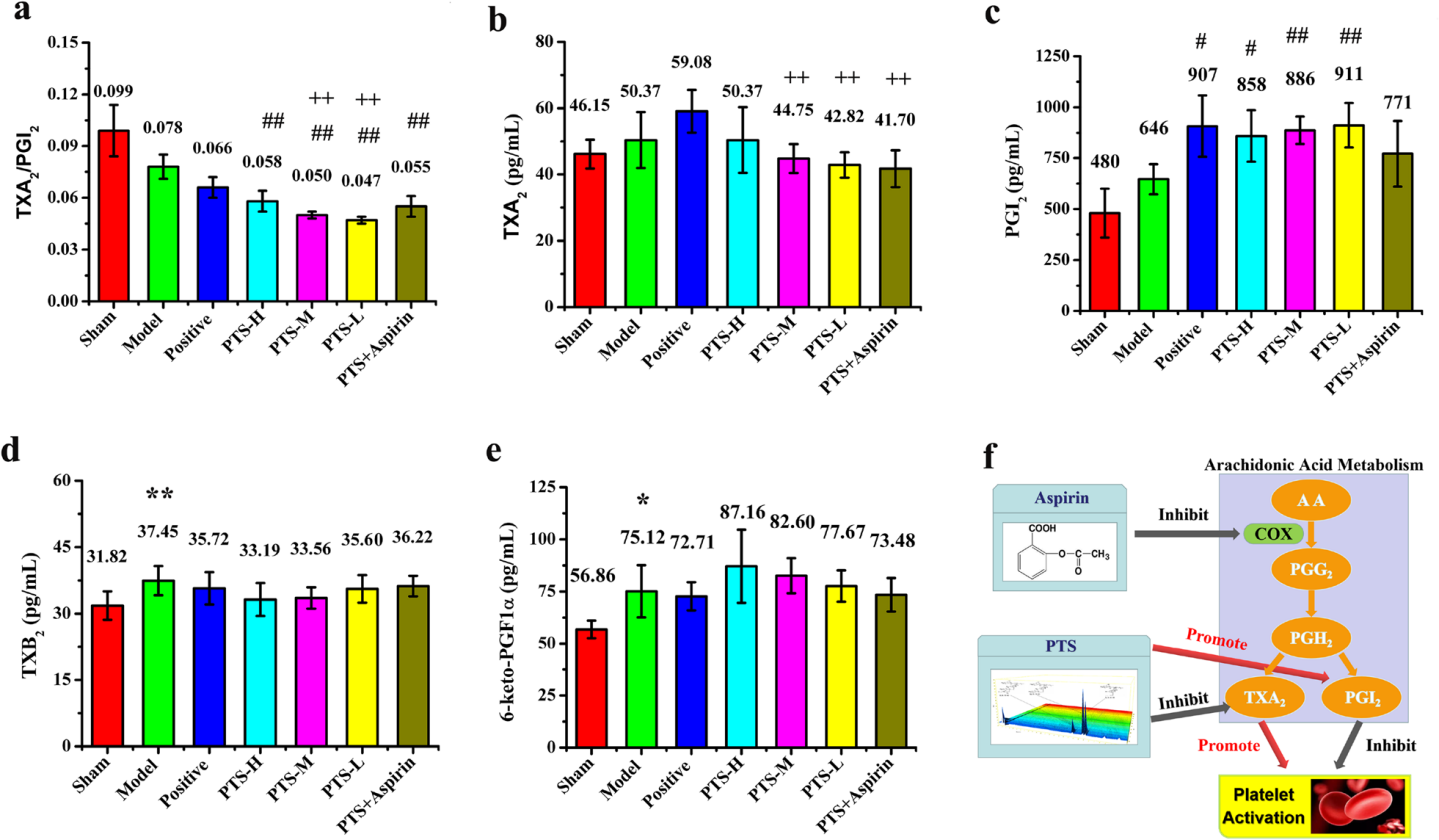
The level of **a** TXA_2_/PGI_2_ ratio, **b** TXA_2_, **c** PGI_2_, **d** TXB_2_, and **e** 6-keto-PGF1α in the serum of each group was determined by ELISA after reperfusion for 22 h. Values are the mean ± SD (n = 8). Comparing model control group with sham surgery group, *P < 0.05, **P < 0.01; comparing treatment groups with model control group, ^#^P < 0.05, ^##^P < 0.01; comparing treatment groups with positive control group, ^+^P < 0.05, ^++^P < 0.01; comparing PTS combined with aspirin group with PTS medium-dose group, ^▲^P < 0.05, ^▲▲^P < 0.01. **f** The different effect of PTS and asprin in arachidonic acid metabolism according to the above data and past researches suggests thatthe main difference between the two drugs is related to an influence on Thromboxane and prostacyclin

PGI_2_ and TXA_2_ are both in vivo metabolites of arachidonic acid, and TXB_2_ and 6-keto-PGF1α are hydrolyzed products of PGI_2_ and TXA_2_. As shown in Fig. 4a–e, following ischemia, the balance of the ratio of PGI_2_ to TXA_2_ in serum, which is an important indicator of AA-induced platelet aggregation, was greatly affected. In the animal experiment, we found that PTS could regulate the TXA_2_/PGI_2_ ratio, which could maintain vascular tension and reduce the risk of gastrointestinal bleeding. When PTS was used alone, PGI_2_ significantly increased compared with that of the treatment group in combination with aspirin. Similar results were also obtained with a reduction in TXA_2_ expression. The increased expression of TXB_2_ and decreased expression of 6-keto-PGF1α also appeared in the PTS/aspirin combination group, and the variation trend of the two metabolites was the same as their prototypical substances.

**Fig. 5:**
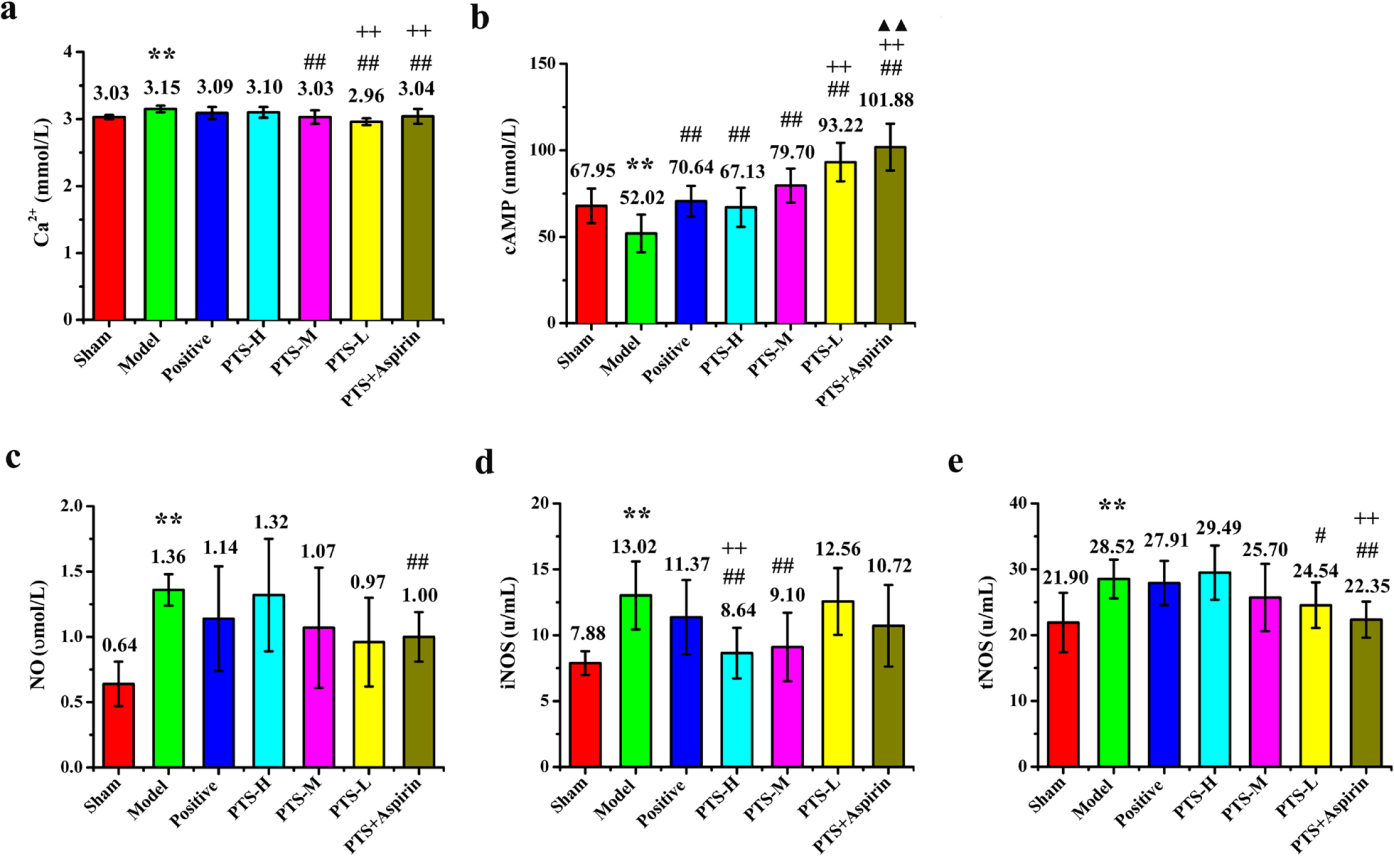
The expression level of **a** Ca^2+^, **b** cAMP, **c** NO, **d** iNOS, and **e** tNOS in the serum of each group was determined by ELISA (cAMP), Microwell plate method (Ca^2+^, NO) and Colorimetric method (iNOS, tNOS) after reperfusion for 22 h. Values are the mean ± SD (n = 8). Comparing model control group with sham surgery group, *P < 0.05, **P < 0.01; comparing treatment groups with model control group, ^#^P < 0.05, ^##^P < 0.01; comparing treatment groups with positive control group, ^+^P < 0.05, ^++^P < 0.01; comparing PTS combined with aspirin group with PTS medium-dose group, ^▲^P < 0.05, ^▲▲^P < 0.01

Conversely, both cAMP and calcium ions are intracellular second messengers and displayed important effects on the formation and exacerbation of platelet aggregation (Fig. 5a, b). The MCAO model reduces cAMP concentration and increases the concentration of free calcium ions in the serum, which could cause platelet activation. Our results revealed that PTS exhibits a dose-dependent effect on cAMP and calcium ions with a slight decrease in the expression of calcium ions and a significant increase in cAMP.

Different concentrations of NO produce protective or toxic effects on blood vessels and the nervous system. Maintaining the concentration of NO within the normal range in the body is of positive significance for regulating platelet activation. In the acute stage of cerebrovascular disease, endothelial NOS and neuronal NOS are induced and their activity would be up-regulated after cerebral ischemia, whereas inducible nitric oxide synthase (iNOS) is activated to produce and release a large number of pathologic inflammatory factors that play an important role in necrosis and apoptosis of neurons. In this study, we found that different doses of PTS could reverse the trend of increasing total nitric oxide synthase (tNOS), iNOS, and NO to different degrees in rats after ischemia/reperfusion, especially in the case of iNOS, which was much lower than the positive control and the combination of aspirin and PTS groups (Fig. 5c–e).

### The regulating effect of PTS on the expression of GP1BA and PAF in the MCAO model

**Fig. 6:**
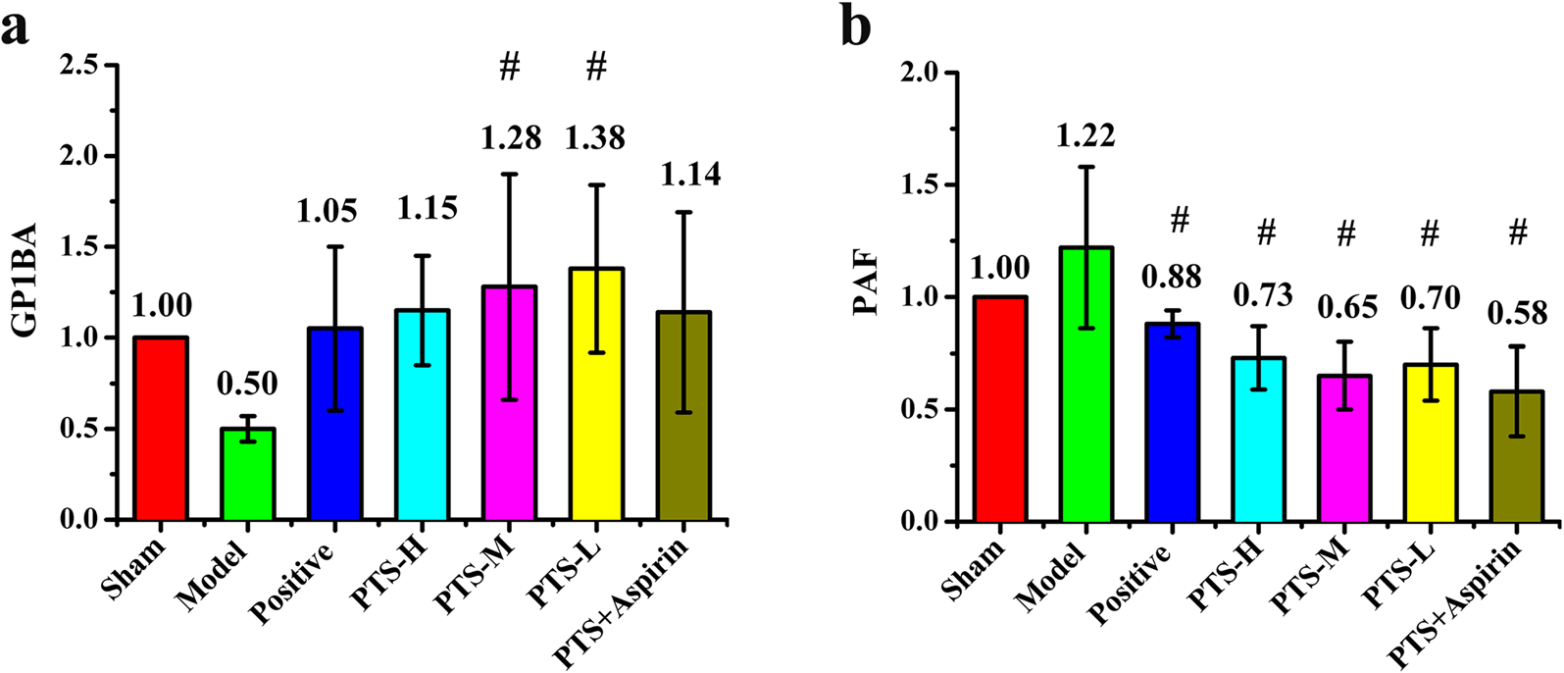
The expression ratio of **a** GP1BA and **b** PAF in each group compared with the sham group determined by Western blot after reperfusion for 22 h. Values are the mean ± SD (n = 3). Comparing treatment groups with model control group, ^#^P < 0.05. An additional figure file shows this in more detail (see Additional file 1)

The expression of GP1BA was markedly decreased in the model group, whereas the expression of GP1BA was increased in the drug treatment groups (Fig. 6a). The effect of PTS on GP1BA protein expression was higher than that of PNS, especially for the low- and medium-dose PTS groups. Meanwhile, compared with PTS alone, the group treated with the combination of PTS and aspirin showed no significant difference. The model group had increased PAF protein expression (Fig. 6b); however, PAF expression was significantly decreased in all of the drug treatment groups (P < 0.05), and the combination of PTS and aspirin group showed the lowest PAF expression, whereas the positive control group showed the highest PAF expression. These results indicated that PTS could upregulate GP1BA protein expression and downregulate PAF protein expression. No previous studies have reported that aspirin had an effect on the expression of GP1BA and PAF, and the results of this study also indicate that no obvious enhancement effect was found by combining PTS with aspirin. Therefore, PTS and aspirin might have different targets and pathways in anti-platelet aggregation.

### PTS binding with GP1BA by SPR test and molecular docking

**Fig. 7:**
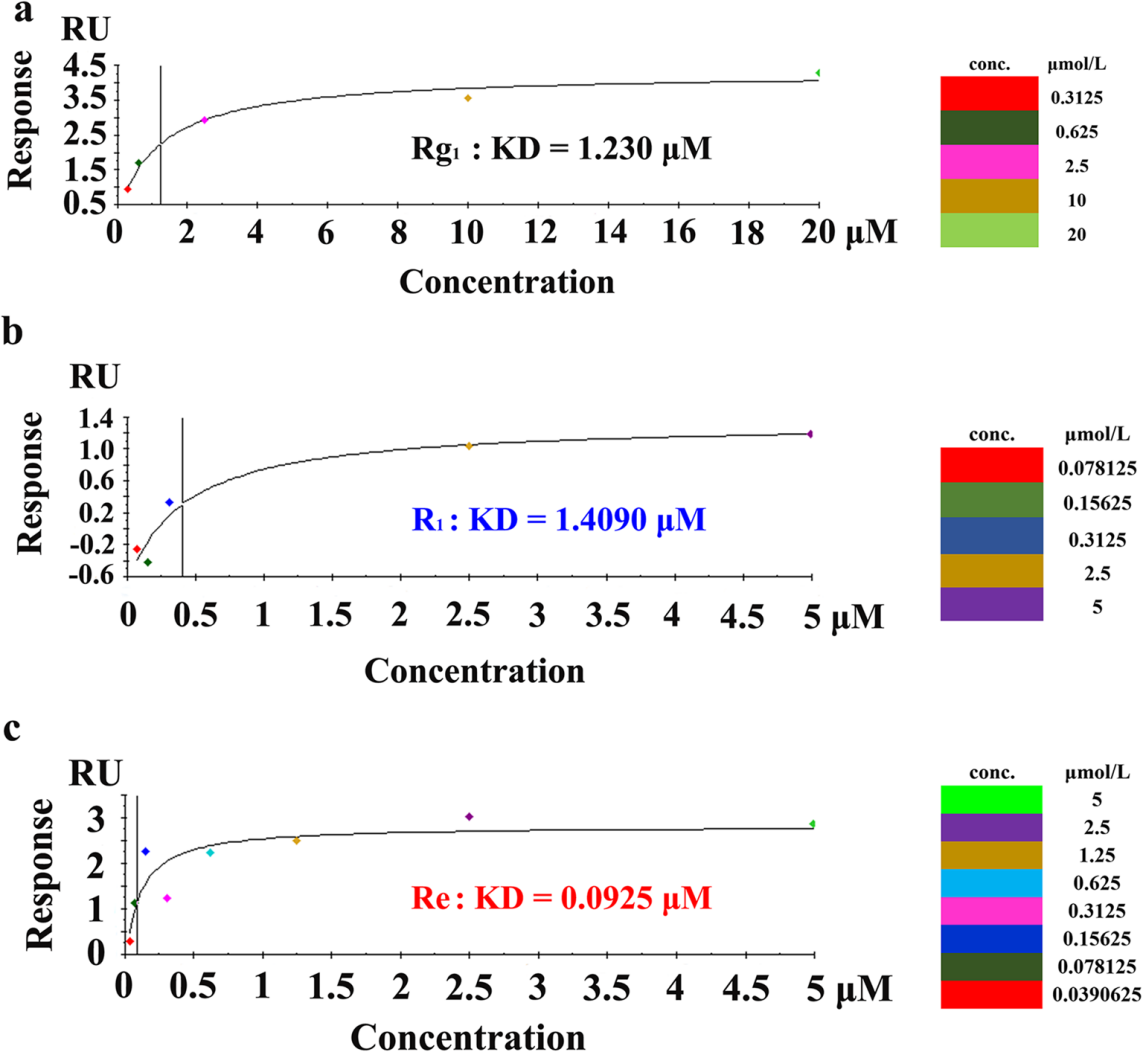
Binding affinity results of PTS with GP1BA. Binding affinity curve of **a** ginsenoside Rg_1_, **b** notoginsenoside R_1_, and **c** ginsenoside Re with GP1BA, respectively. This figure also showed the KD value of ginsenoside Rg_1_, notoginsenoside R_1_ and ginsenoside Re binding with GP1BA

The results of the experiments in the MCAO rodent model suggested that the regulation of GP1BA protein expression might be an important mechanism for anti-platelet aggregation. However, how did PTS regulate the expression of this protein? We used SPR and molecular docking simulation technology to accurately investigate the inter-molecular interactions and molecular recognition between the main monomeric compounds of PTS and GP1BA. We applied multi-cycle kinetics in the experiment. The analytes (ginsenoside Rg_1_, notoginsenoside R_1_ and ginsenoside Re) were prepared by running a HBS-EP buffer containing pH 4.5 sodium acetate. The concentration was set with five gradients, and a blank control with only running buffer was used. The experimental data were fitted by affinity, and all of the KD values of ginsenoside Rg_1_, notoginsenoside R_1_, and ginsenoside Re binding to GP1BA were stated in the μM grade (Fig. 7). All three monomer compounds exhibited high binding activity to the platelet membrane glycoprotein GP1BA. However, the results showed that the KD value of ginsenoside Rg_1_ was higher than that of the other two compounds, which preliminarily suggests that the binding capacity of ginsenoside Rg_1_ might be relatively low. In the SPR test, it was required that the target protein was well-coupled with the chip, and the coupling mode and condition needed to be explored. In this experiment, due to the low isoelectric point of the tested protein GP1BA, the amount of GP1BA protein captured on the CM5 chip was eventually determined to be 987.1 RU using different pH assays. Although this value was not very high, the experiment indicated that the binding amount of the various components of the ginsenosides at different concentrations was different, and the results should be reliable.

**Fig. 8:**
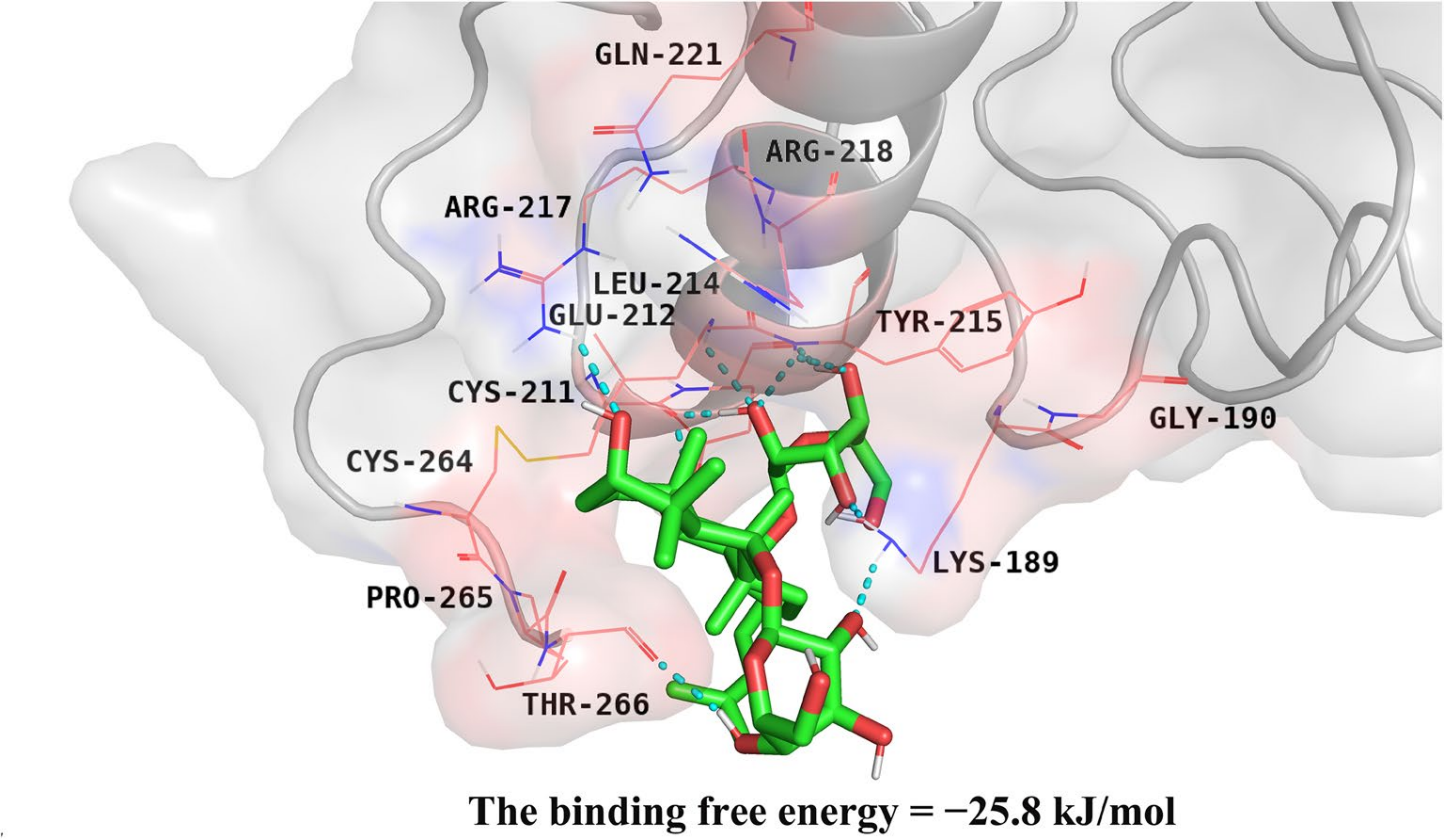
Schematic diagram of molecular docking simulated using PyMOL (The PyMOL Molecular Graphics System, Version 2.3 Schrödinger, LLC). The basic structure of GP1BA is represented by the cartoon in light grey; the protein residues in contact with the ligand are represented by wireframe; the molecule structure of ginsenoside Rg_1_ is represented by a tube, and the blue dots represent the hydrogen-bonding between the above two

The results of the molecular docking analysis indicated that ginsenoside Rg_1_ could form ten hydrogen bond binding sites with the N-terminal amino acid residues of the protein and form a stable structure of the hydrophobic pocket with its surrounding residues. The hydroxyl in ginsenoside Rg_1_ was involved in H-bonding with the Leu^214^, Lys^189^, Cys^211^, Glu^212^, Tyr^215^, Arg^217^ and Thr^266^ residues in the amino- (N-) terminal of the GP1BA protein. Furthermore, ginsenoside Rg_1_ also formed hydrophobic contacts with the Gly^190^, Arg^218^, Gln^221^, Cys^264^ and Pro^265^ residues in the GP1BA protein, which strengthened the bond stability (Fig. 8). The binding free energy (the protein–ligand interaction energy) was − 25.8 kJ/mol, and the Inhibition Constant, Ki was 30.0 μM. The molecular docking conformation of the ginsenoside Rg_1_ and GP1BA proteins could be computer simulated. The results of the affinity and docking energy between the protein and ligand also demonstrated that stable binding could be formed.

According to previous studies [17], one side of the VWF A1 domain was wrapped by the amino N-terminal structure of GP1BA, forming two binding regions linked by the interaction between solvated electrons. The two binding sites of α-thrombin were successively bound to two sites (25 amino acid residues) of the amino N-terminal structure of GP1BA and formed a variety of protein–ligand complexes. This docking study revealed that ginsenoside Rg_1_ also acted at the amino N-terminal of GP1BA, of which ARG-218 was also the common binding site of ginsenoside Rg_1_ and α-thrombin, indicating that ginsenoside Rg_1_ might prevent GP1BA from binding to VWF and thrombin by occupying the amino N-terminal of GP1BA and inhibiting the activity of the GP1BA protein.

## Discussion

As a major cerebrovascular disease, ischemic stroke has become one of the main threats to human health, and now is the second global cause of death [30, 31]. Aspirin was the most widely used anti-platelet agent in acute ischemic stroke treatment and secondary stroke prevention [32]. However, some patients may be allergic to aspirin or cannot tolerate the gastrointestinal bleeding adverse reaction and some other patients may be resistant to aspirin [33]. As a result, these patients have to decrease the dosage of aspirin or refer to other anti-platelet drugs. In recent years, the combination of anti-platelet agents as clopidogrel and aspirin is used regularly in patients with acute coronary syndrome. Medium or low doses of Aspirin and clopidogrel had been recommended in the 2019 American Heart Association/American Stroke Association (AHA/ASA) guidelines [34] to be routinely used for acute ischemic stroke in first 24 h for those patients that did not have received i.V alteplase therapy, but trials also showed a higher bleeding rate with combination therapy [35].

PTS is a quantified dry extract isolated from the root of Panax Notoginseng (Burk.) F. H. Chen. The quality standard of PTS has been included both in Chinese Pharmacopoeia and Deutscher Arzneimittel-Codex/Neues Rezeptur-Formularium (DAC/NRF). PTS has been used clinically in China more than 17 years for IST as agent of anti-platelet aggregation and neuro-protective. PTS showed a good effect in attenuating blood–brain barrier disruption in rats after Ischemia/reperfusion and these effects may associate with multiple actions as anti-inflammatory effects, Shh pathway activation and VEGF, Ang-1 and MMP-9 expression regulation [36, 37]. As revealed in this study of pathological model animal, PTS showed a good therapeutic effect indicated by a decrease in cerebral infarction size, water content in brain tissue, pathological changes in brain tissue, and recovery effect of nerve cells, as well as regulation of related factors and platelet receptor expression in serum. We also found that PTS could regulate the TXA_2_/PGI_2_ ratio, which might reduce the adverse gastrointestinal effects of aspirin, because the loss of the prostacyclin-thromboxane A2 balance was the cause of aspirin's bleeding problems.

Ginsenoside Rg_1_ was the highest content (greater than or equal to 50%) which greatly outnumbered ginsenoside Re, and notoginsenoside R_1_. In addition, a systematic review and meta-analysis proved that ginsenoside Rg_1_ was active ingredient which had a marked efficacy in experimental acute ischemic stroke [38]. In this study, we found that ginsenoside Rg_1_, ginsenoside Re, and notoginsenoside R_1_ all showed high binding activities with GP1BA by molecular interaction test with SPR. As shown in Fig. 1a–c, all of these three main components were dammarane-type tetracyclic triterpenoid saponins and contained the same parent nucleus structure. So it could be concluded by organic structure analysis that the differences in the side chains of each compound had little impact on the docking with GP1BA. Panaxatriol-type saponins are interconverted under the influence of pH value, liver drug enzyme, and intestinal bacteria. For example, ginsenoside Re can be converted into ginsenoside Rg_1_ in vivo [39, 40]. A human pharmacokinetic study demonstrated that the ginsenoside Rg_1_ and notoginsenoside R_1_ could be detected in healthy human blood after oral administration of the Sanqi Tongshu capsules, especially in the case of ginsenoside Rg_1_ with an AUC_0–∞_ and C_max_ in humans that are much higher than those of notoginsenoside R_1_ [41]. Therefore, in this study on molecular docking of PTS and GP1BA, the ginsenoside Rg_1_, which has been confirmed to be fully distributed in the human blood system, was selected as the main focus of our study. Taking the main component ginsenoside Rg_1_ as the representative, the results of molecular docking by computer simulation showed that ginsenoside Rg_1_ and GP1BA could form a stable structure. As mentioned previously for the structural similarity of the major components in PTS, it could also be speculated that other active components and metabolites in PTS might also bind to the GP1BA protein in a similar manner. These studies demonstrated that PTS could reduce VWF-mediated platelet adhesion to vascular endothelium at the site of vascular injury, and also reduce the binding of thrombin to platelets, thereby reducing the probability of platelet aggregation and thrombosis under pathological conditions.

Combined with the results of previous studies [13, 42], it could be determined that the anti-platelet aggregation action of PTS involves early-thrombogenesis through the regulation of GP1BA, along with platelet activation induced by ADP, PAF, AA, collagen and other factors, and activation of intracellular signal transduction, although the influence of PTS on mRNA of GP1BA, how to enhance the expression of GP1BA and related pathways remains to be further studied. Thus, our results provided new scientific evidence of PTS in the clinical application of IS and would be helpful in further research into the mechanism of action and future clinical studies.

## Conclusion

In this study, we found that PTS exhibited a good overall therapeutic effect in ischemia–reperfusion rats after middle cerebral artery occlusion in a dose range of 25–100 mg/kg, which was manifested as significantly reducing the area of cerebral infarction and water content of brain tissue, as well as producing obvious histopathological improvements. By altering TXA_2_/PGI_2_ ratio, activity of nitric oxide-related factors, and second messenger of intracellular signaling cAMP and calcium release in animals, PTS could exert therapeutic effects by regulating relevant neural cytokines and the expression of GP1BA and PAF proteins leading to anti-platelet aggregation. The KD value between all compounds (ginsenoside Rg_1_, ginsenoside Re, notoginsenoside R_1_) and GP1BA presented quite relatively high binding activities, and ginsenoside Rg_1_ and GP1BA could form a stable hydrophobic pocket structure. Furthermore, this study indicates that PTS could competitively inhibit the activity of the GP1BA protein at the level of molecular interactions, implying that PTS reduces the incidence of VWF-mediated platelet adhesion to vascular endothelium at the site of vascular injury and also reduces the binding of thrombin to platelets, thereby reducing the probability of platelet aggregation and thrombosis under pathological conditions.

## Supplementary Information

**Additional file 1.** Figure of GP1BA and PAF in MCAO model by Western Blot.

## Data Availability

The datasets used in this study are available from the corresponding author upon reasonable request.

## Abbreviations

GP1BA: Glycoprotein Ib-α
IST: Ischemic stroke therapy
MCAO: Middle cerebral artery occlusion
PGI_2_: Prostaglandin I2
PNS: Panax notoginseng total saponins
PTS: Panax notoginseng triol saponins
SPR: Surface plasmon resonance
TXA_2_: Thromboxane A2
VWF: Von Willebrand factor
