# Supplementary material for: Anti-platelet aggregation of Panax notoginseng triol saponins by regulating GP1BA for ischemic stroke therapy
Source: Chin Med. 2021 Jan 19;16:12. doi: 10.1186/s13020-021-00424-3 (PMC7816336; doi:10.1186/s13020-021-00424-3)
Supplement: Supplementary file 1 — Additional file 1. Figure of GP1BA and PAF in MCAO model by Western Blot. [file 13020_2021_424_MOESM1_ESM.pdf]

Additional file 1: Figure of GP1BA and PAF in MCAO model by Western Blot

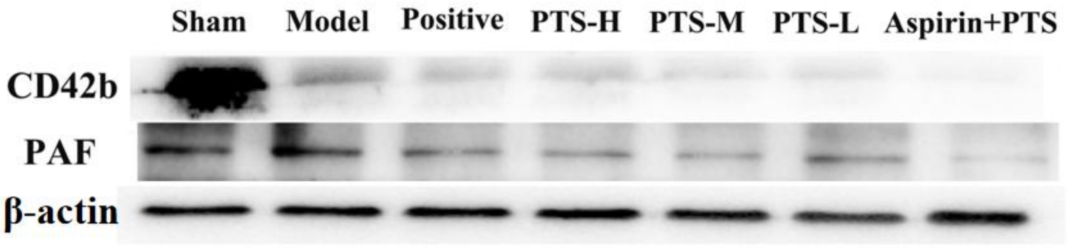

Notes: Effect of PTS on expression of GP1BA and PAF in each group at 22h after reperfusion were determined by Western Blot. β-actin was selected as a reference.
